# Next Generation Imaging Techniques to Define Immune Topographies in Solid Tumors

**DOI:** 10.3389/fimmu.2020.604967

**Published:** 2021-01-27

**Authors:** Violena Pietrobon, Alessandra Cesano, Francesco Marincola, Jakob Nikolas Kather

**Affiliations:** ^1^Refuge Biotechnologies, Inc., Menlo Park, CA, United States; ^2^Essa Pharmaceuticals, Inc. South San Francisco, CA, United States; ^3^Medical Oncology, National Center for Tumor Diseases (NCT), University Hospital Heidelberg, Heidelberg, Germany; ^4^Department of Medicine III, University Hospital RWTH Aachen, Aachen, Germany

**Keywords:** immune topography, solid tumors, immune exclusion, imaging techniques, deep learning, single-cell analysis

## Abstract

In recent years, cancer immunotherapy experienced remarkable developments and it is nowadays considered a promising therapeutic frontier against many types of cancer, especially hematological malignancies. However, in most types of solid tumors, immunotherapy efficacy is modest, partly because of the limited accessibility of lymphocytes to the tumor core. This immune exclusion is mediated by a variety of physical, functional and dynamic barriers, which play a role in shaping the immune infiltrate in the tumor microenvironment. At present there is no unified and integrated understanding about the role played by different postulated models of immune exclusion in human solid tumors. Systematically mapping immune landscapes or “topographies” in cancers of different histology is of pivotal importance to characterize spatial and temporal distribution of lymphocytes in the tumor microenvironment, providing insights into mechanisms of immune exclusion. Spatially mapping immune cells also provides quantitative information, which could be informative in clinical settings, for example for the discovery of new biomarkers that could guide the design of patient-specific immunotherapies. In this review, we aim to summarize current standard and next generation approaches to define Cancer Immune Topographies based on published studies and propose future perspectives.

## Introduction to Cancer Immune Topographies

### Cancer Immunotherapy in Hematological and Solid Tumors

The first application of immunotherapy was reported in 1891, when Dr. William B. Coley saved a patient with inoperable multiple advanced tumors by infecting him with streptococcal bacteria. The immune reaction produced caused shrinkage of the malignant tumor. However, the advent of radiation and chemotherapy resulted in a dismissal of Coley’s approach. This changed in the last decades leading in 2013 editors of the Science magazine to elect cancer immunotherapy as the “Breakthrough of the Year.” In 2018, the Nobel Prize in physiology or medicine was awarded to two cancer immunotherapy researchers, J. P. Allison and T. Honjo, for their discovery of immune checkpoints (1). Today, cancer immunotherapy is considered a promising therapeutic strategy against a variety of hematological and solid malignancies (2–4). Immune checkpoint inhibitors and chimeric antigen receptor (CAR) T-cells can induce durable remissions in many cancers and are clinically accepted as standard treatments for several cancers.

Significant clinical responses have been observed in hematological malignancies, using CAR-T-cells engineered to recognize CD19 molecules on B-cells (2, 5, 6). Treatments with checkpoint blocking antibodies have also been approved by the FDA for melanoma, lung cancer, breast cancer, kidney cancer and many other solid tumor types (7–15). Despite this progress, only a limited subset of patients responds favorably to the treatment and some tumors, such as prostate cancer and most gastrointestinal malignancies, have been proven to be particularly resistant to checkpoint inhibition, particularly when used as single agent (16–19). In general, solid tumors present various challenges to the applicability of immunotherapy, including the selection of the antigen to target, the infiltration of T-lymphocytes into the tumor core and the highly immunosuppressive tumor microenvironment (TME), which are all hallmarks of solid tumors (20). Solid tumors are heterogeneous ecosystems and they can contain different immunological niches in different regions of the same lesion. Systematic documentations of this phenomenon are scarce with the exception of a recent study which used computational image analysis inspired by geospatial data to quantify the heterogeneity of topographies in lung cancer (21). In some types of cancer, such as colorectal cancer (CRC), specific Immune phenotypes are linked to specific genotypes. For example, highly immune-infiltrated tumors are associated with hypermutation, which are mostly due to specific genetic features such as microsatellite instability or mutations in POLD1 or POLE genes in CRC (22).

### Immune Topographies in Solid Tumors: Hot, Cold, and Immune Excluded Tumors

For decades, the gold standard method for diagnosing and subtyping almost any type of solid tumor has been visual examination of histopathology slides of tumor tissue. These slides are unspecifically stained with hematoxylin and eosin (H&E) which allows pathologists to discern cellular and subcellular structures. In spite of the technological progress in molecular biology assays, subjective evaluation of histopathology slides remains the backbone of solid tumor diagnostics. Although H&E staining dyes do not selectively bind to specific cell types, the visual characteristics of different cells allow a reproducible classification of cells into tumor cells, lymphoid immune cells, granulocytes, fibroblasts and other groups of cells in the tumor microenvironment. In particular, tumor-associated lymphocytes (including tumor-infiltrating and peritumoral lymphocytes) can be easily spotted due to their unique size, morphology and staining characteristics. Historically, the presence of such lymphocytes in or around tumor tissue has been regarded as an epiphenomenon of malignant tumor growth. However, mounting evidence supports the notion that the presence of these lymphocytes reflects an adaptive anti-tumor immune response by the host immune system and is a prognostic biomarker as well as a predictive biomarker of response to immunotherapy (23).

**Figure 1:**
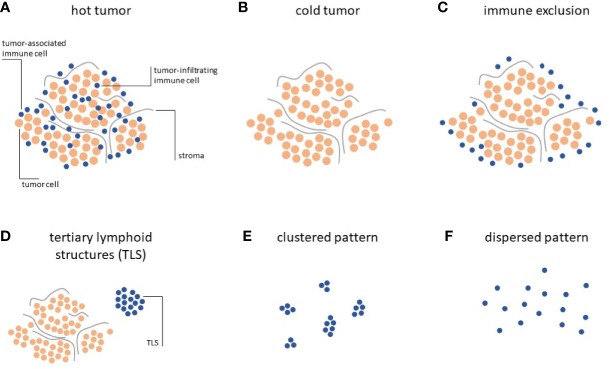
Immune topographies of cancer. **(A–C)** First-order immune topographies. **(D–F)** Second-order immune topographies.

Systematic analyses of the distribution of lymphocytes in histopathology specimen have allowed to classify solid tumors according to three distinct Immune Topographies (24): a) Hot tumors, infiltrated by lymphocytes, i.e. lymphocytes are mixed with tumor cells in the tumor core (**Figure 1A**); b) Cold tumors, characterized by an absence of lymphocytes, i.e. almost no lymphocytes can be seen on histological slides (**Figure 1B**); c) Immune-excluded tumors characterized by an abundance of lymphocytes at the invasive edge of the tumor, but few to no lymphocytes in the tumor core (**Figure 1C**).

Hot tumors present a homogeneous infiltration of T lymphocytes together with the accumulation of pro-inflammatory cytokines, and usually respond better to immunotherapy (25). Immune excluded tumors embody a unique ecosystem, differing from hot tumors, as they display gradients of T-cell exclusion (26). Such gradients are specific to each tumor environment and not present in cold tumors, where T-cells are completely absent. This trichotomy can be detected across most solid tumors and is directly associated with clinical outcome and response to immunotherapy (24, 27–30).

However, few published studies have systematically quantified the trichotomous Immune Topography in solid tumors beyond histopathological description (24). At present, there is only a limited understanding about how cellular mechanisms of immune exclusion may relate to each other in shaping this peculiar phenotype in human cancers, either through converging biological pathways or from a causative standpoint. Compelling data collected through high-throughput analysis would shed light on the spatial and temporal dynamics in which such determinants are involved, allowing for the creation of a harmonized ‘Theory of Everything’ (31, 32). Moreover, mapping the spatial distribution of immune cells in solid tumors also provides quantitative information potentially informative in clinical settings, allowing for the design of combinatorial approaches aimed at improving immunotherapy efficacy.

### Tertiary Lymphoid Structures and Other Second-Order Topographies

Hot, cold and immune-excluded tumors are among the most striking patterns that can be observed in histopathology images. However, in addition to this trichotomy, immune cells in cancer tissue can form additional patterns which have been quantified and linked to biologically and/or clinically meaningful endpoints. These “second-order” Immune Topographies do not rely on pre-defined compartments within the tumor and around the tumor. For example, one such pattern of lymphoid cells observable in histopathology images of cancer is a tertiary lymphoid structure (TLS, **Figure 1D**). These structures are composed of lymphocyte conglomerates organized to resemble lymph node germinal centers and can be observed outside of (33) or within (34) the tumor tissue. The presence and number of these TLS is positively correlated to survival (35) and immunotherapy response (36) in a number of cancer types. However, systematic analyses of association with TLS count and other types of Immune Topographies are still lacking. In summary, it is still not entirely clear how different Immune Topography patterns are correlated to one another and, collectively, to clinical outcome. Large-scale studies in thousands of patients treated with immunotherapy and annotated with clinical outcomes are needed to validate and reconcile these findings in the future. Another approach was recently described by Saltz and coworkers by an unbiased, computational approach to cluster tumors according to their spatial patterns of lymphocyte infiltration (37). In particular, this approach considered the notion of *clustered* (**Figure 1E**) and *dispersed* (**Figure 1F**) lymphocyte infiltration and the authors could demonstrate a link between the observed lymphocyte patterns and patient survival, the ultimate clinically relevant end point. However, this large-scale analysis was limited to a single multicenter dataset, the Cancer Genome Atlas (TCGA), which might suffer from batch effects and other sourcing bias (38).

In this review, we aim to summarize the current knowledge in standard and next generation techniques to define Cancer Immune Topographies, including the performed studies, outcomes and future perspectives.

## Biological Mechanisms of Immune Topographies

### Barriers to an Effective Immune Response in Solid Tumors

Determinants of immune exclusion can be classified into three groups: physical, functional or dynamic barriers. Physical barriers represent a category where T-cells do not come in direct contact with cancer cells, due to mechanical separations. Therefore, activation of the immune effector gene signature is not observed (30). However, T lymphocytes can also engage with cancer cells, but functional determinants block their migration, expansion, function and/or survival within the tumor core. Functional barriers consist of constitutive metabolic interactions among immune cells, cancer cells and cells in the TME. Finally, dynamic barriers include functional barriers, which may be induced only when a contact between T-cells and cancer cells occurs, preventing further infiltration, activation and/or survival of immune cells. Dynamic barriers may not be present in baseline conditions (39–41). Here, we will give a brief overview of these determinants, as it is beyond the scope of this review to describe them in more detail.

### Physical Barriers

Physical barriers include the remodeling of the extracellular matrix (ECM), cancer cell coating factors and changes in vascular accessibility (**Table 1**). In tumor tissues, the most frequent alteration of the ECM includes increased collagen deposition and a rearrangement of its geometry; this leads to cancer-associated fibrosis and possibly to a physical barrier to T-cell penetration (115–120). A variety of chemokines are responsible for this process, which requires the activation of recruited and resident fibroblasts, myofibroblasts, cancer-associated fibroblasts (CAFs) and cancer-associated mesenchymal stem cells (42–44, 121–123). CAFs have also been shown to be responsible for the biosynthesis of CXCL12, which binds and shields cancer cells (64–66).

**Table 1:** Determinants of immune exclusion.

		References
**Mechanical barriers: physical impediments to a direct contact between T cells and cancer cells**		
ECM remodeling	Cancer-associated fibrosis	(42–48)
	Epithelial-to mesenchymal transition	(49–55)
	Filaggrin, desmosomal proteins, endothelin B receptor	(30, 56–58)
Vascular accessibility	HIF-1 and HIF-2 driven angiogenesis	(59–63)
Cancer cell coating	CXCL12	(64–66)
**Functional barriers: determinants limiting migration, function, and/or survival of T cells**		
Metabolic alterations TME	Decrease in amino acids in the TME	(67–72)
	Warburg effect (increase in lactate)	(73–76)
	Increase in extracellular adenosine	(77–80)
	Increase in potassium	(62, 81, 82)
	Altered lipid metabolism	(83)
	Cyclooxygenase and prostaglandin metabolism	(84–86)
	Hypoxia	(87–91)
Soluble factors	VEGF-α	(59, 60, 92–94)
	Cytokines mediated immune suppressive mechanisms	(42, 43, 95–98)
Danger sensing molecules	TAM receptors	(99–102)
	don’t eat me signals	(103–105)
	Tolerogenic cell death/absent immunogenic cell death	(106–108)
Tumor cell signaling	STAT-3, PI3K, NF-κB, Wnt/β-catenin, MAPK, p53	(109–114)
**Dynamic barriers: barriers arising after interaction between T cells and cancer cells**		
	Checkpoint/ligand interactions	(39–41)

Another mechanism involved in the physical exclusion of T-cells may be related to tumor angiogenesis. As cellular proliferation outgrows blood supply, most solid tumors experience hypoxic conditions (124). In hypoxia, genes involved in angiogenesis are upregulated, including the vascular endothelial growth factor family (VEGF) (125, 126). Tumor angiogenesis often produces blood vessels with aberrant morphology and this could result in T-cell exclusion (87, 88, 127). Additionally, VEGF not only plays a prominent role in mediating T-cells infiltration into tumors, but also in regulating their function (59, 60, 92).

### Functional Barriers

Functional barriers consist in metabolic alterations of the TME, immune suppressive soluble factors, danger sensing molecules and tumor cell-intrinsic signaling (**Table 1**). Cancer cell metabolism often leads to the reshaping of TME conditions, depleting it of amino acids (i.e. glutamine), which are essential for proper T-cell function (67, 128–133). Moreover, TME often presents increased concentration of lactate, due to the shift toward glycolytic metabolism of cancer cells (Warburg effect) and increased concentration of ions and other immune suppressive components, such as extracellular adenosine (134–137). Therefore, low pH, low glucose and reduced amino acid presence in the TME collectively lead to T-cell dysfunction.

### T-Cell Signaling

A complex signaling network is responsible for the impaired function of T-cells, leading to lymphocytes that are exhausted, anergic, senescent or presenting stem features. Stem-cell-like T-cells possess the ability to proliferate and persist, but they are unable to mature (81, 138). Recent evidence showed that an overabundance of potassium in the TME triggers suppression of T-cell effector function, while preserving stemness (138). This happens through metabolic remodeling as a result of caloric restriction and a T-cell starvation.

Interestingly T-cell stemness can also enhance the effectiveness of immunotherapy: the generation of CD19-specific CAR-modified CD8^+^ memory stem cells led to long-lasting antitumor response and increased T-cell fitness, in a human acute lymphoblastic leukemia xenograft model (82, 139, 140).

A variety of chemokines have been implicated in the recruitment of T-cells into the tumor nest. In immune excluded tumors, it is possible that gradients of chemokines exist from the periphery to the center. However, additional repulsive mechanisms may limit the propulsion of T-cells, counterbalancing and overpowering attractive signals and reducing the chemo-attractive infiltration. In addition, stressed or dying cancer cells may inhibit proinflammatory signals, thereby affecting the efficiency of the immune response (**Table 1**) (141–143).

### Dynamic Barriers in the Tumor Microenvironment

Finally, dynamic barriers represent a category of impediments absent in baseline conditions, but which arise as a consequence of the interaction between T-cells and cancer cells. This hints to a dynamic crosstalk between the two, at early stages. An example is the inducible activation of PDL-1 triggered by the production of IFN-γ by T-cells (**Table 1**) (39–41).

It is unclear if a predominant biology is responsible for most immune excluded cases or if an indiscriminate contribution of factors could better explain this complex phenomenon. Moreover, at present studies have not investigated if a correlation exists between immune exclusion mechanisms and tumor type or stage.

## How to Quantify Immune Topographies

### *In Vivo* and *Ex Vivo* Approaches

Over the years, studies have demonstrated the existence of a plethora of determinants playing a role in the immune excluded phenotype. Modern high-throughput techniques allow us to create pan-cancer Immune Topographies, characterizing spatial and temporal distribution of T-cells in the TME (24). Mapping *ex vivo* immune cells and correlating such distributions with determinants of immune exclusion and morphological parameters, would provide mechanistic insights into the dynamic organization of factors responsible for this phenomenon. It is possible that specific determinants of immune exclusion could correlate with some tumor types or with the tumor stage, rather than appearing randomly and chaotically.

*In vivo* studies offer information to design effective personalized combinatorial immunotherapies and clinical monitoring. Finally, it may be possible to integrate the data obtained from *in vivo* and *ex vivo* techniques, for the different determinants of immune exclusion. Such a comprehensive analysis might lead to the understanding of a common biology at the basis of the immune excluded phenotype.

### Histology Images

Histopathology images are a versatile and well established method to analyze the tumor microenvironment and Immune Topographies in solid tumors. Histological specimens are routinely generated from preclinical tumor models and are available for almost any patient with a solid tumor in the clinic. The standard staining method for histopathology slides is hematoxylin and eosin (H&E) which allows for a rough classification of cells in the TME. By visually looking at histopathology slides or digitized whole slide images, pathologists can quantify patterns of antitumor immune response.

Although hot, cold and immune-excluded Immune Topographies can be visually determined just by looking at H&E stained histopathology slides, two methods have enabled a more quantitative description of these topographies: Immunohistochemistry and computer-based analysis.

### Immunohistochemistry and Multiplex Imaging Techniques

Immunohistochemistry (IHC) methods can be used to selectively label certain immune cell subtypes in histology images, allowing for a more nuanced definition of Immune Topographies. IHC uses antibodies which are directly or indirectly coupled to certain dyes, allowing it to highlight markers specific to certain cell types. For example, cytotoxic lymphocytes are defined by a presence of the CD8 protein on their cell membrane. IHC methods have recently inspired a whole range of more sophisticated multiplex methods, allowing to characterize the expression of multiple proteins in one image. Multiplex fluorescence imaging (144), multiplex brightfield imaging (145) are among the most widely used methods to deeply characterize tumor-associated immune cells in a spatially resolved way. However, these methods are much more expensive, time-consuming and complex than H&E staining and usually require access to specialized and costly equipment. Thus, the advantages of these deeper methods need to be balanced against the simplicity of classical H&E histopathology, which allows for a broader characterization of thousands of patient samples in larger cohorts. Accordingly, most studies which have analyzed H&E histopathology images include a much higher number of patients than studies adopting more specialized methods.

### Hypoxia-Associated Proteins

As previously mentioned, hypoxia is a key player in the immune excluded phenotype. Hypoxia is responsible for a dramatic reshaping of cellular transcriptional programs, through the activity of specific transcription factors called Hypoxia Inducible Factor 1 and 2 (HIF-1 and HIF-2). These proteins are responsible for the upregulation of a subset of genes essential to ensure adaptation, survival and proliferation in hypoxic conditions (146–148). Common hypoxic markers used in IHC include: CAIX, VEGF-A, EPO, GLUT-1 and GLUT-3, osteopontin, BNIP3, PDK1, LDHA, and LOX (149–153). These proteins are transcriptionally induced by HIF-transcription factors. HIF-1 can also be directly assessed in IHC, but correct sample handling is essential. The half-life of HIF-1 at 20% oxygen is approximately 5 min while other markers (i.e. VEGF-A and CAIX) are more stable (147, 154–156). Therefore, it is crucial to select the most appropriate proteins to test, based on sample processing procedures. Exogenously administered compounds can also be used to detect hypoxic regions in IHC. A nitroimidazole molecule called pimonidazole and a pentafluorinated derivative of the 2-nitroimidazole etanidazole, called EF5, are the most widely used (157–159). These non-toxic compounds are administered from a few hours to 48 h prior to biopsy and immunochemical detection is then performed. Pimonidazole directly correlates with the severity of hypoxia, and IHC has been successfully used to assess tumor hypoxia in patients with cervical carcinoma, prostate cancer and head and neck carcinoma (150, 157, 160). EF5 is also routinely employed to detect gradients of hypoxia as shown in studies on patients with head and neck tumors and uterine cervix cancer (161, 162). EF5 has also been used to detect hypoxia in atherosclerotic plaques in mice and in xenograft models of human colorectal carcinoma and sarcoma (163, 164).

### Digital Pathology Approaches

Digital pathology, i.e. computer-based processing of digitized histopathology slides, has been used to automatically detect and count tumor-associated immune cells. Such approaches can be used to automate detection of Immune Topographies and to establish quantitative thresholds for classification of a given sample in either class. In the early days of digital pathology, the sheer size of scanned whole slide images (WSI) has been an obstacle for many researchers to analyze such data. Nowadays, however, more widespread availability of digital slide scanners, cheaper storage media and the emergence of easily usable open source software such as QuPath (165) enables almost any researcher at academic institutions to use computer-based approaches for quantitative analysis of pathology slides. These quantitative analyses can be performed with H&E stained images, single-immunostained images or multiplex images. One way of quantifying hot-cold-excluded topographies by means of digital pathology is with the “Immunoscore” (166), which has been extensively validated in large-scale studies (28). While the original “Immunoscore” protocol relies on proprietary software marketed by a commercial entity, the underlying principle can be re-built based on publicly available information (167).

### Deep Learning-Based Image Analysis

Medical image data, and particularly digitized histopathology slides contain a large amount of information which is not entirely used. In particular, human observers, who visually analyze histopathology slides cannot objectively and quantitatively extract all relevant information. Deep learning is a method from the realm of artificial intelligence, which in recent years has revolutionized computer-based image analysis in non-medical and medical domains alike. Specifically, when applied to digital whole slide histology images, deep learning can extract biologically and clinically relevant information. In particular, immune-related features can be extracted from histopathology images. For example, gene expression signatures of cancer-infiltrating immune cells can be detected solely from H&E images in multiple types of solid tumors (168, 169).

### Genome Sequencing Technologies

A comprehensive mechanistic insight regarding the correlation of functional, physical and dynamic barriers with morphological parameters in human cancer could be achieved thanks to last advances in high throughput analysis. These techniques are laying the foundation for a pan-cancer comprehension of the complexity of factors orchestrating the immune response in the TME. Recent studies profiled the TME of several cancer types including lung cancer, hepatocellular carcinoma, medulloblastoma, melanoma, head and neck cancer, pancreatic cancer and glioma (170–176).

Current developments in automated, multiplexed platforms to detect biomarkers and the increasing number of spatially resolved profiles of transcripts and metabolic products, could be important to provide an integrated landscape of molecular determinants driving the phenomenon of immune exclusion. Obtaining correlations between markers of different types of barriers, degree of immune infiltration/activity and tumor type/stage would allow us to investigate if one or multiple pathways are prevalent in human cancer or if these pathways just overlap indiscriminately. Such information, accumulated in a large cohort of human cancers, would be pivotal to improve diagnostic strategies and to predict the response to treatments.

Transcriptional profiling of “immune-mediated, tissue-specific destruction (TSD)” events led to the creation of an immune signature called immunologic constant of rejection (ICR) (31, 177–179). ICR is a 20-gene signature and characterizes a convergent pathway leading to TSD, also called immune rejection. Such signature can be observed in a variety of immune events: tumor regression, autoimmunity, clearance of pathogens and allograft rejection (179). ICR expression was correlated with a better prognosis in breast cancer patients and was validated as a prognostic predictive parameter in a pan-cancer cohort of patients treated with an anti-CTLA4 immune checkpoint inhibitor (109, 180, 181). The tumor inflammation signature (TIS) is considered as another immune predictive biomarker and it is an 18-gene signature (182, 183). TIS has been shown to be enriched in patients responding to anti-PD1 treatment and the expression patterns were conserved among tumor types (182).

Bioinformatics studies have been performed during the years, to investigate immune signatures in different types of tumors. These analyses rely on data from public cancer databases and provide a coarse evaluation of T lymphocytes functional status in bulk tumor samples. Computational deconvolution analysis on bulk RNA-seq data can be used to infer infiltrating cell types. Such analysis is limited by the existence of specific gene signatures, relative to cell types (184–187). However, no spatial or temporal resolution can be obtained from bulk bioinformatic studies. In order to achieve more detailed information about T-cell populations within the tumor mass and their functional state, single-cell techniques were developed and experienced tremendous progress in the past few years.

### Single-Cell RNA Sequencing

Single-cell RNA sequencing (scRNA-seq) allows the investigation of the expression of hundreds of genes in a single experiment, enabling systematic identification of cell populations in a tissue. This technique provides insights into tumor heterogeneity and it has been used to assess both abundance and functional state of tumor associated cell types (188–193). ScRNA-seq has been increasingly employed due to a reduction in costs (sequencing and cell isolation) and improvement in throughput.

The most common scRNA-seq technologies rely on microfluidic devices which use patterned microwells for single-cell isolation or aqueous droplets in a continuous oil phase. Once isolated, cells are lysed and a whole transcriptome approach can be performed (i.e. Smart-seq2, MATQ-seq, SUPeR-seq) (194–196). Alternatively 3’-end or 5’-end sequencing technologies are available (i.e. Drop-seq, CEL-seq, Seq-Well, MARS-seq, Chromium, Quartz-seq, DroNC-seq, STRT-seq, etc.) (197–206). These two categories present different transcript coverage and each protocol has specific features, benefits and drawbacks.

Unfortunately, single-cell techniques require monodisperse cells, leading to loss of spatial information during cell isolation. Moreover, tumor solid biopsies could lead to biases and failure to identify the whole transcriptional profile or the truthful tumor-associated cell infiltration landscape. This can be due to the sampling location and to the fact that, only a small fraction of cells from the biopsy, can be sequenced (207–209). LCM microdissection is a method of sample dissection which consents the preservation of spatial information and partially rescues the technical limitation mentioned above. Cells from the region of interest are collected using laser pressure catapulting or through a near-IR laser, after being microscopically identified (210–212). Experimental or computational spatial reconstruction can also be obtained *via* immunohistochemistry, laser scanning microscopy, fluorescent *in situ* RNA sequencing (FISSEQ) or with anchor genes, through single molecule fluorescence *in situ* hybridization (smFISH) (213–217).

Single cell analysis allowed the investigation of the heterogeneity of tumor cells and tumor-infiltrating immune cells in breast cancer, clear cell renal cell carcinoma, colorectal, glioblastoma multiforme, melanoma, liver, ovary, non-small-cell lung carcinoma, nasopharyngeal cancer, squamous cell carcinoma of the head and neck and gastric cancer (168, 188–192, 218–222). The immune cells prevalently identified in most of these tumors were T lymphocytes; however myeloid cells, B cells and natural killer cells were also present at lower frequencies.

Immune cell subtypes were transcriptionally characterized, and their ontogeny was mapped, together with cell trafficking to different tissues or tumors (223). Clonotype expansion and migration was monitored by barcoding V(D)J recombination at the T-cell receptors (TCR) and B cell receptors (BCR) loci (224–226). V(D)J recombination occurs during T and B cell maturation, resulting in the diverse repertoire of TCR and BCR present in the lymphocyte population. Gene expression signature of T-cell clusters reflects a specific functional status and such diversity is crucial in clinical settings, to predict immunotherapy response. Indeed, T-cell populations with lower exhaustion levels were associated with a better prognosis in a variety of different cancer types (227–229). A study on gastric cancer reported that the interferon regulatory factor-8 transcription factor (IRF-8) was downregulated in CD8+ tumor infiltrating lymphocytes (TILs), leading to an exhausted phenotype. Patients with lower IRF-8 levels in CD8^+^ lymphocytes tended to have worse prognosis (230). A recent publication showed that, in early-stage triple-negative breast cancer, among CD8^+^ TILs, tissue resident memory cells display high expression of cytotoxic molecules, inhibitory checkpoint and genes associated with proliferation. This study suggests that T-cell exhaustion is a gradual process (231, 232). In clear cell renal cell carcinoma, PD-1 was found widely expressed in both CD8^+^ and CD4^+^ T-cell populations, while other inhibitory molecules were present only in a subset of PD-1 positive T-cells (189). Studies on the functional state of T-cells were also performed in melanoma and non-small-cell lung carcinoma patients, allowing the characterization of dysfunctional T-cells in the TME (191, 233). Xiao et al. (222) developed a computational pipeline to investigate the metabolic landscape of the tumor, at single cell resolution. They analyzed metabolic gene expression profiles of more than 9000 single cells from melanoma and squamous cell carcinoma of the head and neck. Metabolic pathways in tumor cells were found to generally be more plastic and, interestingly, glycolysis and oxidative phosphorylation both correlated with hypoxia at the single cell level. Metabolic features of immune cells were also identified and found to be altered. Characterizing the metabolic landscape in the tumor core could provide insights into the organization and prevalence of functional barriers.

Interactions among cells play a central role in shaping the TME altering cell metabolism, immune response and creating barriers to lymphocytes infiltration or activity (234, 235). Despite the study of cell interactions using single cell approaches is at early stages, a new publicly available repository of curated receptors, ligands and their interactions is available. CellPhoneDB (www.cellphonedb.org) takes into account the subunit architecture of both ligands and receptors and, coupled to scRNA-seq data, is a powerful tool to infer cell-cell communication networks (236, 237). Recently, scRNA-seq coupled to CellPhoneDB has been used to reveal interactions between Th2 and mesenchymal cells, in asthmatic human donors (238). ProximID is another strategy to create a cellular network based on scRNA-seq data. ProximID can be used to discover new niches interactions in different tissues, *via* microdissection of small interacting cell clusters and inference of the cell types present in the dissected entity through scRNA-seq. Boisset et al. (239) used ProximID to study mouse bone marrow and found specific interactions between megakaryocytes and mature neutrophils and between plasma cells and myeloblasts and/or promyelocytes. Moreover, they identified stem cell interactions in small intestine crypts.

Single cell analysis is not confined to the investigation of the transcriptome and, recently, the combination of multiple measurements (DNA, RNA, proteins) has been suggested as a comprehensive strategy to understand the TME complexity (223, 240). Innovative techniques such as G&T-seq and DR-seq allow to sequence both DNA and RNA, from single cells (241, 242). Genomic DNA and full-length mRNA are captured and physically divided before amplification and library preparation. These techniques, despite allowing for the comparison of gene expression data and corresponding genomic data in the same cell, increase the risk of sample loss or contamination and present a moderate reduction in coverage distribution.

Another combination of multi-omics techniques, which provides information about the transcriptional status of cells, consists of coupling ATAC-seq with RNA-seq. ATAC-seq can be considered a technique to assess genome-wide chromatin accessibility (243–245) and it relies on a genetically engineered hyperactive Tn5 transposase (246). Such transposase allows fragmentation of chromatin and integration of NGS adapters into open chromatin regions (247, 248). ATAC-seq coupled with RNA-seq was used to identify potential gene regulatory regions in glucagon-secreting α-cells and insulin-secreting β-cells (249) and to unravel disruptions of transcriptional regulations and gene expression in lung cancers (250).

Quantification of proteins and mRNAs simultaneously in individual cells can be obtained through different methodologies: Cellular Indexing of Transcriptomes and Epitopes by Sequencing (CITE-seq), RNA Expression and Protein Sequencing (REAP-seq) and Antibody sequencing (Ab-seq) (251–254). The workflow includes the creation of a pool of barcoded antibodies againsT-cell surface proteins of interest. Then, cells bind to barcoded antibodies and are encapsulated within a droplet, as single cells. Finally, the scRNA-seq libraries are prepared and sequenced. Such an approach overcomes the lack of correlation that sometimes is found between mRNA and protein levels, providing a more accurate characterization of the cellular phenotype.

The integration of different layers of information could be pivotal to provide insights into signaling networks regulating the immune excluded phenotype. However, as mentioned before, a primary drawback of single cell techniques is the loss of spatial information, which occurs during sample processing. In order to create systematic Immune Topographies, it is of crucial importance to characterize spatial and temporal distribution of lymphocytes in the TME. Therefore, technologies permitting simultaneous transcriptional assessment and preservation of tumor morphology or restoration of spatial information are preferable.

### Spatial Transcriptomic Methodologies

**Figure 2:**
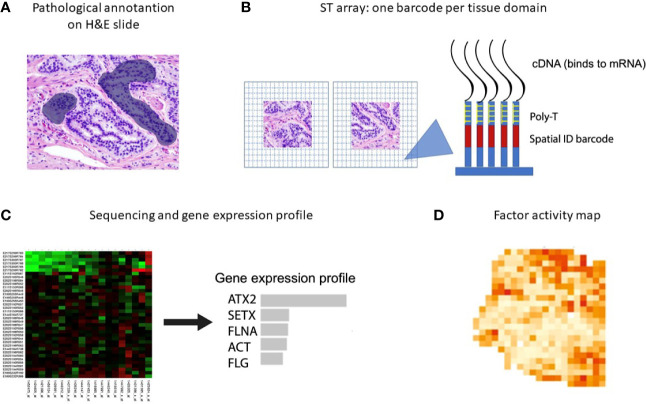
Spatial transcriptomics workflow including the downstream analysis. **(A)** Histological tumor sections are annotated by a pathologist and sections of interest are stained with hematoxylin and eosin before permeabilization. **(B)** The sections are placed on glass slides containing RT-primers arrayed as spots that correspond to tissues domains. The RT-primers at each spot have a unique spatial ID barcode, which is sequenced along with the transcript to enable trace-back to a specific tissue domain. **(C, D)** After sequencing, gene expression profiles and factor activity maps are created.

New spatial transcriptomic (ST) methodologies exploit spatially barcoded oligo-deoxythymidine microarrays, allowing for unbiased mapping of transcripts (**Figure 2**) (255). ST has been performed to investigate prostate cancer, gingival tissue, breast cancer, pancreatic ductal adenocarcinoma, melanoma, adult human heart tissue, mouse, human and mouse spinal cord tissue and mouse olfactory bulb (221, 256–262). ST does not provide a high resolution as each area resolves the transcriptome of 10-200 cells (~ 100 µm), depending on the tissue context.

**Figure 3:**
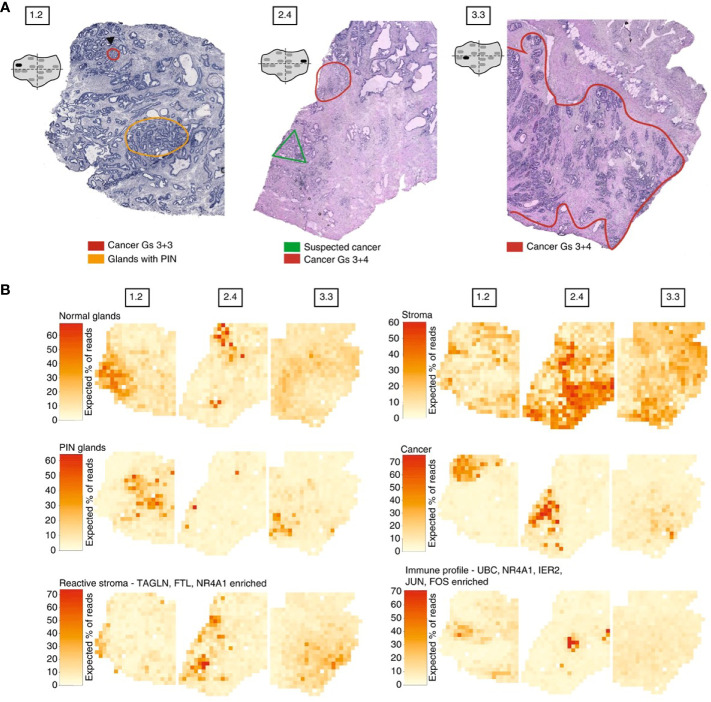
Example of spatial transcriptomic analysis on three prostate cancer biopsies: histology and gene expression factors (257). **(A)** Annotated brightfield images of tissue sections of interest, stained with hematoxylin and eosin. **(B)** Factor activity maps for morphological features (normal glands, PIN glands, stroma and cancer cells) and for inflamed regions (reactive stroma, immune profile).

Moncada et al. combined microarray-based ST with scRNA-Seq generated from the same sample, to identify enrichments of specific cell types and subpopulations across spatially-defined regions of pancreatic tumors (221). Berglund et al. assessed the transcriptomes of nearly 6,750 tissue regions through ST from a patient with prostate cancer. They extracted different expression profiles for stroma, immune cells, and cancer cells (**Figure 3**) (257). Another example is Thrane et al., who applied the ST technology to melanoma lymph node biopsies. The transcriptomes of over 2,200 tissue domains was sequenced, revealing a detailed landscape of melanoma metastases (259). Nanostring technologies recently developed a high-plex panel to be used with the GeoMx™ Digital Spatial Profiler (DSP). This panel includes more than 1,400 genes to spatial profile tumor and immune pathways, including checkpoint inhibitors, intrinsic cancer cell pathways and predictive markers (263).

ST protocols usually achieve a quite low resolution, however an implementation of this technique called Slide-seq can spatially resolve maps of histological sections at 10 µm resolution (264). Slide-seq substitutes the barcoded oligo-deoxythymidine with DNA-barcoded beads, harboring probes to trap the RNA. This technique was performed to map individual cell types, physically and functionally, in brain cryosections.

Other spatial techniques of interest include fluorescence *in situ* hybridization (FISH), NICHE-seq technology and spatially-resolved transcript amplicon readout mapping (STARmap). FISH allows to achieve a highly multiplexed single-molecule visualization of transcripts. In particular, multiplexed error-robust single-molecule fluorescent *in situ* hybridization (MERFISH) enables RNA imaging of individual cells using physically imprinted error-robust barcodes for individual RNA species. Subsequent rounds of imaging allow to measure these barcodes (265–269). Xia et al. (266) measured RNA species from ∼10,000 genes in different subcellular compartments. He also observed transcriptionally distincT-cell states and revealed spatial patterning, in U-2 OS cultured cells.

**Figure 4:**
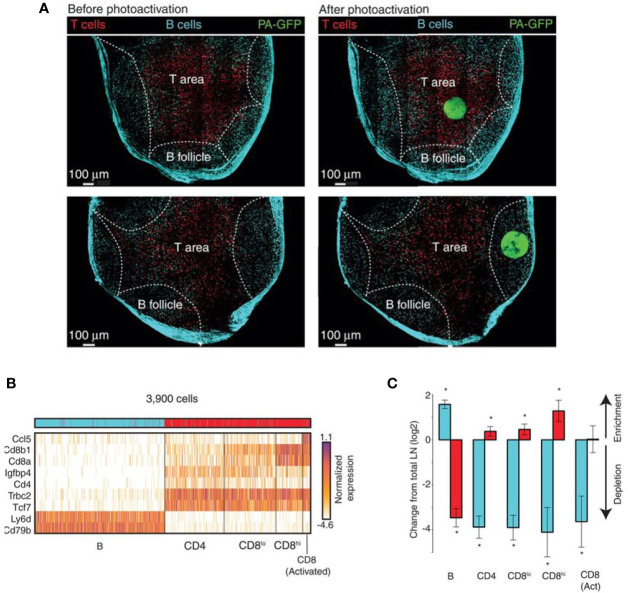
Example of NICHE-seq, assessing the cellular composition of defined niches (215). **(A)** Two-photon laser scanning microscopy (TPLSM) images of naïve inguinal lymph nodes from PA-GFP host mice before and after photoactivation (green). In red, adoptively transferred cells and cyan marks the T-cell area and the B follicles, respectively. **(B)** Expression profile from photoactivated B follicles (cyan) or T-cell areas (red). **(C)** Relative enrichment of different T-cell types [(B), CD4, CD8^low^, CD8^high^ and CD8 Activated] in each subregion. *p < 0.05.

NICHE-seq technology allowed isolation and sorting of immune cells from a visually selected niche in model animals, expressing a photoactivatable green fluorescent protein (215, 270). ScRNA-seq was performed on sorted cells. This technique preserves the cell states and allows the investigation of the TME influence on immune cells. NICHE-seq was performed to identify T and B cells in mouse lymph nodes and spleens, after virus infection (215). It also revealed niche-specific expression programs and changes in immune localization, in melanoma and naïve inguinal lymph nodes in mouse models (**Figure 4**). However, due to the two-photon laser scanning microscopy which is required to perform this technique, application of NICHE-seq is currently limited to preclinical research.

Three-dimensional distribution of transcripts in mouse model was achieved *via* STARmap. This technique integrates hydrogel-tissue chemistry, targeted signal amplification, and *in situ* sequencing (271, 272). STARmap was used to map 160 to 1,020 genes in 3D-intact tissue from brain mice. It successfully revealed molecularly defined gradient distribution and clustering of neuron subtypes (271).

### Emerging Technologies for Multiplexed Molecular Profiling of the TME

The development of targeted therapies that may be efficacious in reprogramming the host immune response to recognize and eliminate tumor cells requires accurate identification of the various inflammatory cells and the special relationships between them within the TME. While currently established techniques enable routinely interrogation for up to two protein markers and evaluate their expression by visual examination, there is a growing need to reliably query many more targets (including both proteins and mRNAs) simultaneously in a given tissue specimen, in order to more precisely characterize the TME within and between tumors. Three new technologies (i.e not based on IHC or IF platforms) aimed at achieving these goals, including multiplexed ion beam imaging (MIBI), codetection by indexing (CODEX) and digital special profiling (DSP) are discussed below.

Multiplexed Ion Beam Imaging (MIBI) is a new technology platform, based on the CYTOF technique that preceded it, with the capability to detect and visualize a large number of protein *in situ* using secondary mass spectrometry to image antibodies tagged with isotopically pure elemental reporters (273). In contrast to standard multiplexed IHC protocols, sample preparation involves one-step, rather than sequential, application of a cocktail of elementally tagged antibodies (274). Samples are subsequently subjected to a rasterized oxygen duoplasmatron primary ion beam which liberates the lanthanide adducts of the bound antibodies as secondary ions and recorded by a TOF-MS. For each physical pixel in the analyzed tissue a mass spectrum is recorded and reflects the abundance of the queried antigens in that location. Recent publications (275) showed that the high-parameter capability, sensitivity and resolution of MIBI are well suited to understanding the complex tumor immune landscape including the spatial relationships of immune and tumor cells and expression of immunoregulatory proteins.

Co-Detection by Indexing (CODEX) is another novel technology for highly multiparametric *in situ* analysis of protein expression using tissue sections. One of the benefits of this technique is its use of a standard fluorescence microscope rather than an ion bean coupled to a mass spectrometer. But unlike the other platforms, published reports involving CODEX have only utilized frozen tissue cut onto glass slides rather than formalin fixed, paraffin embedded (FFPE) tissue sections. Like the other multiplexing methods, multiple antibodies are applied to a single tissue section for visualization simultaneously; however, the antibodies are tagged with unique DNA oligonucleotides, rather than fluorophores or rare metal elements and then crosslinked to their cellular targets. The process typically involves a single step of immunostaining with up to 40 antibodies each label with a distinct oligonucleotide tag. Visualization of a tissue-bound antibody requires specific PCR-based extension of each antibody bound oligo followed by annealing to a complementary strand of DNA coupled with specific fluorophore. This process is totally automated. However, since the analysis typically involves imaging two to five DNA tagged antibodies at the time a complete analysis of a single tissue requires approximately 30 h to image a 1 cm^2^ at 400 nm resolution. At the end of the multicycle rendering protocol each group of antibodies is visualized at a known predefined cycle of the indexing protocol and the multiplexed image is reconstructed (276).

The above mentioned techniques all use antibody-based methods to detect antibody-protein complexes in a tissue section. By contrast, digital special profiling (DSP) is a technology platform which allows, in a non-destructive manner, to profile multiple proteins and RNA from a wide variety of samples types including FFPE tissue sections (263, 277). Briefly, the method uses antibodies or mRNA probes coupled with UV photocleavable oligo tags for the digital detection of specific proteins and transcripts, respectively. After probe hybridization to slide-mounted tissue, UV light exposure is used to liberate the oligo-tags within a small predefined region of interest (ROI) (278–280). The probes are then automatically collected and quantified on a standard nCounter systems (for up to 800-plex profiling or mRNAs or proteins) or sequenced on NGS platform (potentially for unlimited multiplexing) and counts are mapped back to the tissue location, thus producing a spatially resolved digital profile of analyte (protein or mRNA) abundance within each ROI (278–280). Since the UV light is projected into the sample using two digital micromirror devices containing one-million semi-conductor-based micromirrors, a complete flexibility in the pattern of light utilized for high-plex digital profiling of the tissue can be reached. This mechanism results in diverse, automatically configurable, ROI profiles including 1) tumor only; 2) tumor microenvironment only; 3) unique cell types and rare cell features; 4) spatial gradient around cell features; 5) simple hand-selected geometric areas or a combination of the above methods (277). Furthermore the technology does not destroy the sample thus allowing for multiple profiling cycles of the same tissue section or subsequent DNA sequencing of the same section.

Reports of application of this technology to immuno-oncology clinical trial samples are emerging (278, 279). Immuno-oncology clinical trial samples examined using DSP have already provided key insights into the mechanism of action of combination therapy in melanoma (278, 279). While such sophisticated approaches to tissue evaluation of biomarkers hold tremendous promise they are nonetheless in their infancy and therefore come with one or more caveats at this time including costs, lack of standardization across labs, time and labor intensive protocols and lack of widespread availability.

### *In Vivo* Imaging and Functional Imaging

*In vivo* imaging of T-cell distribution could be a powerful strategy to provide dynamic and spatial information regarding immune exclusion in tumors, during preclinical studies and in a clinical setting. Non-invasive cell tracking would allow us to monitor and quantify cellular delivery and effectiveness of immunotherapeutic approaches. A robust technique would also allow the selection of an appropriate dosing regimen. Over the years, significant developments in imaging immune cells were made and a variety of techniques is currently available for preclinical or clinical use.

Optical detection includes fluorescence or bioluminescence imaging and is mainly performed in preclinical settings, due to its limited depth of penetration. However, numerous whole-body techniques are routinely used in health care and can also be a valid tool to monitor immune cell kinetics: positron emission tomography (PET), single photon emission computed tomography (SPECT), computed tomography (CT) and magnetic resonance imaging (MRI). These techniques require in-vitro or in-vivo labeling of T-cells (281). During in-vitro labeling, cells are harvested, processed, and then infused back into the model organism or patient. Labeling procedures can be classified into two types: direct and indirect (282). Direct labeling is easy to perform and radiotracers, MRI-based contrast agents or fluorophores are internalized by the immune cells. This technique does not allow long term monitoring of cells as mitotic events result in the dilution of the signal. Tracer uptake, retention capacity and changes in cellular features, due to the internalization of the probe, are further drawbacks of direct labeling (281, 283).

Indirect labeling requires genetic modification *via* stable transfection of cells with a reporter gene such as luciferases or fluorescent proteins, which do not require an additional tracer. Other reporter genes such as sodium iodide symporter (NIS), or herpes simplex virus–thymidine kinase (HSV-TK), require further probes for imaging. This approach is preferred for long term imaging because the reporter gene is inherited, but genetic manipulation raises safety concerns (284–287).

*In vivo* labeling occurs directly in the organism and requires the injection of radiolabeled antibodies into the body, to target immune cells. A two-step approach has also been developed, where bispecific antibodies, containing a binding domain for the epitope and one for the tracer, are injected into the organism. Labeled probes are then injected, in order to bind the previously administered antibody. This method allows the use of safer isotopes, with a faster radioactive decay (281, 282).

### Optical Detection Techniques

Intravital microscopy includes a variety of approaches that allow one to distinguish individual cells from tissues and, therefore, investigate immune cell kinetics *in vivo* (288, 289). Optical probes allow for repeated scanning of tissues, providing a spatial and temporal dimension to cellular interactions. For example, intravital microscopy enabled visualization of the dynamic interactions between cancer cells and immune cells in the TME (290). It has also been used to decipher the behavior of B-cells and T-cells in germinal centers of lymph nodes (291).

Currently, the two main tools for intravital microscopy are the confocal microscope and the multiphoton microscope. Despite their potential for high-resolution and low phototoxicity, optical detection techniques are used exclusively in preclinical studies because of their low penetration depth (1–2 mm) and risk of photobleaching. Confocal microscopes have reduced costs but increased autofluorescence and scattering, therefore the imaging depth is in the range of 20 to 50 µm (289, 292). Tavri et al. (293) used fluorescence microscopy to track fluorophore-labeled, tumor-targeted natural killer cells to human prostate cancer xenografts.

In contrast, multilaser scanning microscopy uses tunable titanium-sapphire lasers that operate in the near infrared range (NIR), allowing for superior tissue penetration (200 – 300 µm). Increased imaging depths (500 µm) can also be obtained in brain and cleared tissues (294, 295). Two-photon laser scanning microscopy (TPLSM) requires simultaneous excitation by two photons with longer wavelengths than the emitted light. This particular type of excitation suppresses the background noise and reduces photobleaching. Moreover, using NIR for excitation also minimizes scattering in the tissue (296–298).

**Figure 5:**
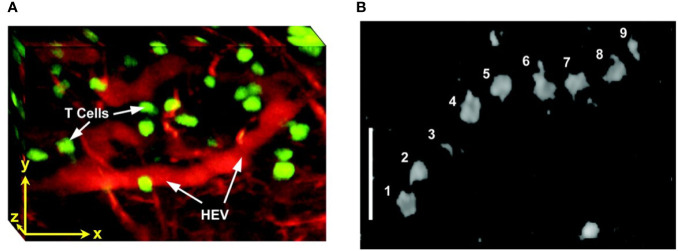
Intravital two photon imaging of naïve T-cells in lymph nodes (302). **(A)** 3D reconstruction representing 85 x 120 x 75 μm of the T-cell area. Fluorescently labeled T-cells (green) are observed in the proximity of presumptive high endothelial venules (red), identified by i.v. injection of tetramethylrhodamine dextran. Scale bar 30 μm in all axes. **(B)** Video-rate imaging of a T-cell flowing in a small vessel within a T-cell region of the node. Image is a superposition of nine consecutive video frames and shows progression of a single labeled T-cell traveling at about 0.03 cm/s within a blood vessel. Scale bar 25 μm. *Copyright (2003) National Academy of Sciences, U.S.A*.

TPLSM was used to visualize the effects of anti-CD19 CAR-T treatment on intracranial primary central nervous system lymphoma (PCNSL), in the same animal over weeks (291). CAR-T-cells infiltrated the tumor inducing regression of PCNSL and increased long term survival. Multiphoton intravital microscopy was also used in lymph nodes, to show import dynamics of the nuclear factor of activated T-cells (NFAT) in the cells. A fluorescently labeled NFAT reporter was used in combination with the nuclear marker histone protein histone 2B (H2B) (299). Stoll et al. (300) developed a protocol for extended four-dimensional confocal imaging of T-cells and dendritic cells, reporting dynamic visualization of antigen-specific T-cells interacting with dendritic cells within intact explanted mouse lymph nodes. Two-photon laser microscopy was also performed to investigate the dynamic behavior of individual T-cells within intact lymph nodes (301, 302) (**Figure 5**). Finally, Bousso et al. (303), performed real-time analysis of the cellular contacts made by developing thymocytes undergoing positive selection in a three-dimensional thymic organ culture.

Bioluminescence (BLI) enables long-term cell tracking, through reporter enzymes such as firefly luciferase, renilla luciferase or bacterial luciferase (304, 305). BLI has higher sensitivity than fluorescence imaging due to the absence of autofluorescence generated by excitation light. BLI and multilaser scanning microscopy were successfully adopted to investigate small populations of T-cells: less than 10.000 cells in live animals (306). Chewning et al. (307) created a novel transgenic mouse model for *in vivo* tracking of CD4^+^ T-cells, using a human CD2 mini-gene to direct luciferase expression specifically to T-cell compartments. Kim et al., used BLI to show that tumor-specific T-cells upregulate IL-2 expression in hypoxic conditions in a model of human B cell lymphoma (308). BLI was also used to track migration of immune cells to sites of inflammation (306, 309).

Cerenkov luminescence imaging (CLI) is based on the detection of visible photons emitted by Cerenkov radiation. Cerenkov luminescence is emitted when a charged particle traverses a dielectric medium with a velocity greater than the phase velocity of light in the medium (310–312). CLI has been performed to optically monitor the biodistribution of ^32^P-ATP labeled T lymphocytes, in small rodents, *in vivo* (313, 314). Results were comparable to those obtained with radioluminescence imaging and T-cell localization in the tumor mass was definitively confirmed by flow cytometry (313).

## Clinically Applicable Detection Techniques

### Digital Histopathology

Histopathology slides are available for almost any patient with a solid tumor, but immune topographies are currently not assessed in clinical routine. While subjective visual examination of tissue slides can be used to roughly quantify, computer-based approaches are ultimately much more scalable and objective. In several countries, digitization efforts for routine histopathology are underway (315). Once this digital infrastructure is established, development and refinement of histopathology-based Immune Topography biomarkers could be accelerated and in turn, clinical rollout of these biomarkers would be markedly facilitated.

### Magnetic Resonance Imaging

MRI is a routinely used, non-invasive diagnostic technique that provides soft tissue contrast with high anatomical resolution. It is considered safer than PET, as it does not use ionizing radiation. Drawbacks of MRI include high instrumentation costs and relatively low sensitivity (316, 317).

MRI can complement PET imaging, co-registering soft-tissue anatomy and multimodal imaging for T-cell tracking is becoming more common. Multimodal imaging allows different imaging methods to be combined simultaneously, providing a multi-layered and complete information regarding the dynamics of immune cells (281, 318, 319).

Standard MRI is based on the detection of signals emitted by protons (^1^H) that are part of the water present in human tissues. Due to the molecular composition of tissue, absorption of a specific electromagnetic impulse generates signals of different intensities. In addition to ^1^H, MRI can be performed on other isotopes such as ^31^P, ^15^N, ^13^C, ^23^Na and ^19^F (317, 320). In some cases these methods are considered less efficient because of the low abundance of these chemical elements *in vivo*, leading to poor signal intensity. ^19^F MRI is gaining more interest as a tool to investigate cell behavior, driven by advances in MRI technology and scan protocols. Indeed, ^19^F MRI provides images with high signal-to-noise ratio and current ^1^H MRI instruments require minimal hardware upgrades to acquire ^19^F-based images (321, 322). However, ^19^F MRI has a detection limit of approximately 10^3^ – 10^5^ cells per voxel *in vivo* (323).

Magnetic nanoparticles (i.e. iron oxides, gadolinium and manganese chelates) can label cells by entering the cytoplasm, binding to the membrane or to reporter proteins. Labeled immune cells have been used to monitor cell interactions *in vivo* and to dissect immunological processes in deep tissue areas. One of the most extensively used nanoparticles in the study of T lymphocytes behavior is superparamagnetic iron oxide (SPIO) (324, 325). The long-term recruitment of cytotoxic T-cells to tumors was studied using a dextran coated SPIO particle, derivatized with a peptide sequence from the HIV-tat protein (326). Wu et al. have developed negatively charged superparamagnetic iron oxide (PAsp‐PCL/SPIO) nanoclusters to monitor the migration of dendritic cells into lymphoid tissues *in vivo* and correlated this with immunotherapy results in mice (327). Tremblay et al., used CD8^+^ cytotoxic T-cells, regulatory T-cells and myeloid derived suppressor cells labeled with SPIO particles, to monitor the efficacy of DepoVax in mice implanted with HPV-based cervical cancer (328).

Cell labeling probes, based on perfluorocarbon nanoemulsions paired with ^19^F MRI detection, have also been widely used to monitor immune cells. Fluorine-dense perfluorocarbon (PFC) nanoemulsions display hydrophobic and lipophobic characteristics and have been engineered to be endocytosed, even by non-phagocytic cells in culture (329). Commonly used PFC include perfluoropolyether (PFPE), perfluoro-15-crown-5-ether (PCE) and perfluorooctyl bromide (PFOB) (323, 330). Despite a lack of evidence supporting the exocytosis or degradation of PFCs, once internalized in the cells, mitotic events lead to the dilution of the signal limiting long- term studies. Nanoparticles are usually cleared by the reticuloendothelial system, in particular from the Kupffer cells in the liver (331, 332).

Chapelin et al. used ^19^F MRI to monitor CAR-T biodistribution and immunotherapy efficacy on immunocompromised mice bearing subcutaneous human U87 glioblastomas (333). Another example includes the study performed by Gonzales et al., whereby T-cells were labeled with PFC *in vitro* and their distribution detected by ^19^F MRI *in vivo*, in melanoma-bearing mice (334). A clinical trial was performed in 2014 to investigate the use of a PFC nanoemulsion in the detection of immunotherapeutic dendritic cells delivered to colorectal adenocarcinoma patients. Composite ^19^F/^1^H overlay images were created and showed that, despite the lack of treatment efficacy, ^19^F MRI enabled visualization of injected cells in patients using a clinical scanner within acceptable scan times (335).

### Immuno-PET

Immuno-PET is a sensitive and non-invasive method used to investigate immune cell interactions in clinical settings, allowing quantification of T-cell dynamics. Indeed, immuno-PET can quantify viability and retention of T-cells in the primary tumor mass and secondary lesions, which may provide insights into immunotherapy efficacy. Clinical imaging could be used to monitor steps of T-cell proliferation, trafficking and infiltration and give insights into mechanistic aspects of the process and effectiveness of induced T-cell response. Although PET and SPECT possess excellent signal-to-noise ratio and unlimited depth penetration, they provide limited anatomical information (336, 337).

Immuno-PET combines antibody specificity against immune cells with PET, which uses radioactive tracers to visualize human tissues. Antibodies recognizing specific features of immune cells are coupled with radioactive isotopes such as ^11^C, ^18^F, ^68^Ga, ^44^Sc, ^64^Cu, ^89^Zr, ^124^I. Radionuclides need to be covalently bound to the antibody and remain kinetically and thermodynamically stable, in order to obtain good quality images. Therefore, their chemical properties and half-lives are fundamental parameters to consider when designing a study (338, 339).

**Figure 6:**
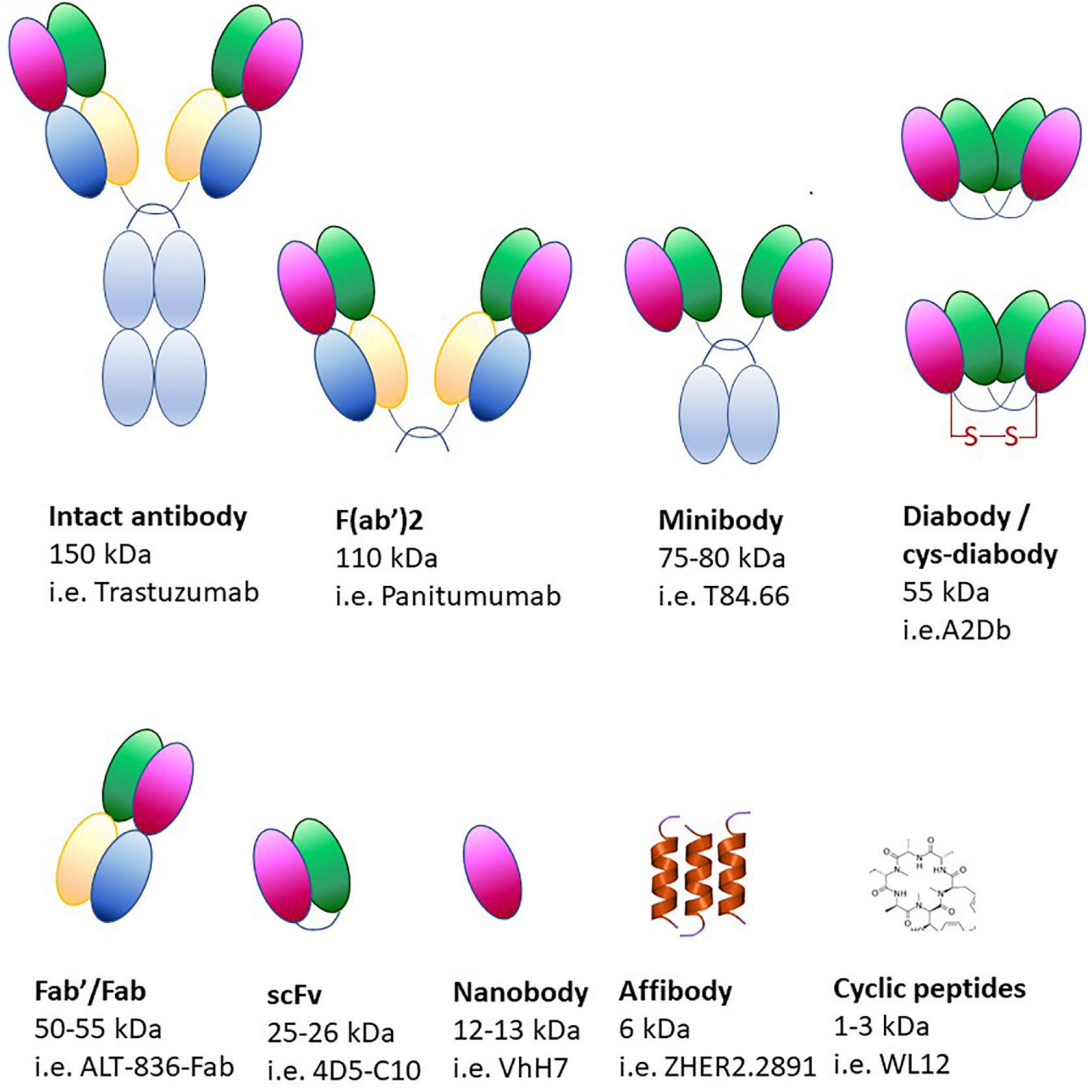
Protein-based scaffolds for targeting cell antigens *in vivo*.

Using monoclonal antibodies in immuno-PET produces images of optimal quality but, due to their size (~150 kDa), it can take up to a week to reach the imaging site after injection (**Figure 6**) (340, 341). Due to slower circulation and clearance times, radionuclides with longer half-lives are required for the labeling of monoclonal antibodies (^89^Zr, ^124^I). Although radionuclides can provide information over long periods of time, they constitute a biohazard as patients are exposed to higher radiation doses. Moreover, the size of monoclonal antibodies exceeds the clearance cut-off value of glomerular filtration (60 kDa), therefore their clearance occurs in the liver, thus precluding its imaging. Smaller molecules have been developed over the years (minibodies, diabodies, single-chain variable region fragments, nanobodies, affibodies), which still retain the specificity of the antibodies and have more desirable pharmacokinetic properties and deeper tissue penetration (**Figure 6**).

Adverse reactions to non-human antibodies are rare but they comport a safety risk for the patient, therefore it is pivotal to ensure that tracking antibodies have minimal pharmacological or toxicological effects. To minimize the risk of adverse reactions, antibodies of camelids, cartilaginous fish or human are often used (342, 343). Camelid antibodies are significantly smaller than standard antibodies and only consist of IgG heavy chains (344). Smaller antibodies can be conjugated with shorter-lived nuclides such as ^18^F, ^64^Cu, ^44^Sc and ^68^Ga. Antibody fragments contain only the targeting and binding components with their sizes ranging from 7 to 100 kDa. Another category includes affibodies, which are constituted of three alpha helices (~6–7 kDa) resulting in high contrast PET images that can be obtained within hours of their administration (339, 345, 346).

Diabodies labeled with ^89^Zr or ^64^Cu were used in specific targeting of CD4^+^ and CD8^+^ receptors resulting in targeting of T lymphocytes *in vivo* (347, 348). Tavare et al. showed that engineered anti-CD8^+^ minibodies were applicable for immuno-PET imaging of endogenous CD8^+^ T-cells in a murine model system (349). Nanobodies were also used to investigate the distribution of intratumoral CD8^+^ T-cells and CD11b^+^ myeloid cells in a colorectal mouse adenocarcinoma model. Response to anti-PD-1 treatment was assessed and showed the difference in CD8^+^ and CD11b^+^ cells infiltration, in responding and non responding tumors. Only the tumors that were completely infiltrated by CD8^+^ T-cells went into full remission (350). Larimer et al., synthesized an anti-CD3 imaging agent labeled with ^89^Zr, to predict tumor response to anti-CTLA-4 treatment in a murine tumor xenograft model of colon cancer. Higher presence of CD3^+^ TILs, revealed by an increased uptake of the radiotracer, correlated with responsive tumors (351).

Reporter genes provide another strategy to target antibodies for immuno-PET. Reporter genes are transfected or transduced into cultured cells and they encode for a protein specifically targeted by the radiolabeled tracker. While genetic manipulation is considered a biohazard, modern gene-editing technologies have developed safe harbor locations and reduced the risk of mutagenesis. Reporter genes have been used to monitor cell-based immunotherapies in preclinical and clinical studies (338, 352, 353). In a study with glioblastoma patients, cytotoxic T lymphocytes (CTLs) were engineered to express the herpes simplex virus type 1 thymidine kinase (HSV1-tk) alongside a glioblastoma-targeting interleukin-13 zetakine (353, 354). Immuno-PET coupled to MRI provided information regarding the locations of CTLs in the glioblastomas together with detailed anatomical context (353). The expression of a foreign protein can be recognized by a patient’s immune system, causing adverse reactions. To avoid an immune response against the foreign reporter protein, endogenous human reporters have been designed. However, these probes trade off immunogenicity for reduced contrast. The sodium iodide symporter (NIS) is considered one of the most promising reporters for preclinical and clinical studies (284, 355, 356). Endogenous expression of NIS is confined primarily to salivary and lacrimal glands, lactating mammary glands, the thyroid and stomach. NIS probes have been used to monitor immune cells, viral vectors, oncolytic viruses, tumor cells and cellular therapies by PET and SPECT in both animal models and patients (284).

Antibodies engineered for use in immuno-PET can target a variety of epitopes on T lymphocytes. When naïve T-cells are activated, several cell surface markers are upregulated. However, expression of these markers does not imply, per se, cytotoxic effector functions. Metabolic activity is modified in active T-cells including glycolysis and the upregulation of nucleic acid metabolism. Therefore, specific enzymes involved in the metabolic pathways constitute good tracking candidates. Finally, the T-cell effector function can also be targeted (318, 338).

Surface markers used for activated T-cells include interleukin-2 receptor (IL-2R), OX40 (CD134), TCR complex and co-receptors CD3, CD4, and CD8. Activated T lymphocytes present high levels of IL-2R on their surface and several studies investigated the distribution of such immune cells *via* IL-2 labeling, with PET and SPECT.

For example, in primary melanoma [99mTc]Tc-HYNIC-IL-2 accumulation was observed at metastases. ^18^F-labeled IL-2 was developed as a PET tracer and its uptake was shown to increase upon tumor treatment (357, 358). Clinical trial NCT02922283, completed in February 2020, aimed to study the biodistribution and kinetics of the tracer ^18^F-FB-IL2 in patients with metastatic melanoma. Results have not yet been published. The activation marker OX40 has also been targeted and imaged with PET using a specific ^64^Cu-conjugated murine antibody (359). Humanized OX40 agonist monoclonal antibodies are currently being tested in early phase clinical trials.

T-cell receptors (TCR) present a constant membrane turnover that leads to internalization and accumulation of the anti-TCR probe. Studies have tracked human T-cells using ^89^Zr labeled anti-mouse TCR. A highly sensitive imaging approach was proposed by Klar et al., targeting the TCR2.5D6 on T-cells, which recognize a peptide expressed on leukemia cells (360). T-cell co-receptor CD3 was targeted to monitor anti-CTLA-4 treatment in colon cancer xenograft mouse models. Anti-CD3 antibody labeled with ^89^Zr was used to quantify T-cell infiltration revealing that tumor regression correlated with high levels of infiltration (351). Anti-CD4 and anti-CD8 labeled with ^89^Zr or ^64^Cu have been used to monitor T-cells in mice and human (361–364). Moreover, anti-CD8 minibodies are currently under investigation in clinical trials (NCT03107663, NCT03802123, NCT03610061).

Cell surface markers may present different expression patterns during the progression of the disease therefore, to obtain more comprehensive information, multiple markers should be tested concomitantly.

Metabolic changes during T lymphocyte activation can be monitored with labeled probes including amino acids, hormones, sugars or nucleosides (**Table 2**). As previously mentioned, activated T-cells switch to glycolytic metabolism and upregulate the intake of substrates.

**Table 2:** Markers for T-cells imaging.

Category	Name	Reference and clinical trials
**T cell surface**	Interleukin-2 receptor alpha chain (CD25)	NCT02922283 (357, 358, 365–367)
	OX40 receptor (CD134)	NCT02318394 (359, 368)
	T cell receptor (TCR)	(361, 369, 370)
	CD3	(351, 371, 372)
	CD4	(362)
	CD8	NCT03107663, NCT03802123, NCT03610061 (347, 363, 364)
**Metabolic pathways**	L-leucine analogue	(373–375)
	^18^F-FAC	(376–378)
	^18^F -CFA	NCT03409419 (377, 379–381)
	^18^F-FLT	(382–384)
	^18^F-F-AraG	NCT03311672, NCT03142204, NCT03007719 (385, 386)
	^18^F-FDG	(387–391)
**Effector function**	Granzyme B	(392–395)
	Interferon-gamma	(396)
	Checkpoint receptors	NCT03065764, NCT02760225, NCT03313323 (397–399)

Fluciclovine ^18^F-FACBC, an analog of L-leucine, is a radiolabeled amino acid that is imported into activated T-cells due to upregulation of the amino acid transporter ASCT2 and LAT-1 (373–375, 400, 401). Labeled substrates for enzymes involved in the deoxyrubonucleoside salvage pathway have been developed such as ^18^F-FAC, ^18^F -CFA, ^18^F-FLT, and ^18^F-F-AraG (318).

^18^F-FAC is a labeled deoxycytidine analog, with high-affinity for the enzyme deoxycytidine kinase (dCK). This tracer is being investigated in an early phase clinical trial (NCT03409419), that is recruiting patients with metastatic melanoma and who are undergoing TIM-3 targeted immunotherapy. ^18^F-CFA is also a substrate for dCK and is studied as a potential cancer biomarker for treatment stratification and monitoring. ^18^F-FLT is a thymidine analog that is trapped intracellularly due to its phosphorylation by thymidine kinase 1. It is used to monitor T-cell activation and cancer cell proliferation in medical practice (376, 379, 382, 383).

Arabinosyl guanine is a molecule with specific toxicity to T lymphoblastoid cells and T-cells. AraG prodrug has been used in patients with T-cell acute lymphoblastic leukemia and T-cell lymphoblastic lymphoma (402). Engineered AraG leads to the development of a ^18^F-F-AraG probe that is retained by primary T-cells and it is a substrate for deoxyguanosine kinase. Such a tracker could provide information about T-cell dynamics in the TME and other pathologies involving the immune system (337, 385). A clinical trial to assess ^18^F-F-AraG biodistribution in cancer patients who are expected to undergo immunotherapy or radiation therapy is currently recruiting (NCT03142204). A recent study demonstrated that ^18^F-F-AraG PET imaging could be used to report immune activation *in vivo*, in mice with rheumatoid arthritis (403).

Finally, the metabolic tracer ^18^F-FDG measures the rate of glycolysis in active T lymphocytes, due to the upregulation of the glucose transporters GLUT isotypes. However, tumor cells also present increased rates of glycolysis, therefore imaging can lead to false positive signals (338, 387, 388). The increase in substrate uptake is similar between cancer cells and T-cells, therefore metabolic radiotracers often lead to difficulties in image interpretation.

In order to specifically monitor active T lymphocytes, probes targeting their effector functions have been developed (**Table 2**). For example, granzyme B, released by CD8^+^ T-cells and natural killer cells, is considered one of the main mechanisms through which T-cells mediate cancer cell death (404). A recent study tested the probe [^68^Ga]Ga-NOTA-GZP that targets murine or human granzyme expression. Imaging made it possible to differentiate responders from non-responders, within immunotherapy-treated mice (392). The human probe showed high specificity in human samples, revealing a candidate predictive biomarker for cancer immunotherapy (392). Interferon-gamma (IFN-γ) is a pleiotropic molecule implicated in immune surveillance, playing a role in pro-apoptotic and antitumor mechanisms. However, evidence points to a protumorigenic role for IFN-γ in downregulating major histocompatibility complexes and upregulating checkpoint inhibitors. Although clinical trials assessing the efficacy of anti-cancer therapies based on IFN-γ reported limited success, IFN-γ-mediated response is still correlated with a positive patient prognosis (405). Gibson et al., used an ^89^Zr-labeled anti-IFNγ probe to predict immunotherapy response after HER2/neu vaccination in mouse mammary tumors. Immuno-PET demonstrated that IFN-γ levels in situ, after vaccination, were inversely correlated with tumor growth rate (396). Therefore, targeting soluble cytokines by immuno-PET could be an interesting strategy to quantify immune response directly *in situ* and predict the response to immunotherapy.

## Outlook

### A Comprehensive Biological Theory of Immune Phenotypes in Solid Tumors

A current major challenge in immunotherapy is the increase of its therapeutic potential in solid tumors. Preclinical research demonstrated the existence of a variety of determinants that play a role in shaping the TME, affecting immunotherapy response. The dynamics and distribution of these factors probably change during time and may also vary according to tumor type.

Further studies are required to understand the spatial and temporal distribution of mechanisms involved in the immune exclusion phenomenon and their interdence. Modern techniques allow high-throughput analysis of immune cell distribution *ex vivo* and *in vivo*. It would be interesting to correlate tumor stages with degree of immune infiltration and determinants of immune exclusion, in a pan-cancer investigation. This correlation would provide information on the mechanism(s) of immune exclusion, allowing to integrate the different determinants into a unified ‘*Theory of Everything*.’ Moreover, a comprehensive analysis would also provide insights into approaches that should be adopted in order to improve the efficiency of immunotherapy and the rationale for innovative translational combinatorial treatments.

### Clinical Translation of Immune Topographies

Ultimately, scientific insight into Immune Topographies in solid tumors could lead to a benefit of cancer patients. In particular, determining Immune Topographies at baseline (before starting a systemic treatment such as immunotherapy or chemotherapy) could inform physicians about the chances of treatment response. Thus, Immune Topographies could help to choose one of several available treatment options for a given patient. In addition, dynamically observing Immune Topographies during treatment might enable oncologists to adjust treatment accordingly. Compared to other biomarkers in oncology, Immune Topographies are intuitively understandable, linked to biological processes of demonstrated relevance and are comparatively easy to measure. However, clinical implementation will depend on larger-scale retrospective analysis and prospective clinical trials evaluating the utility of Immune Topographies for managing cancer treatment.

## Author Contributions

All authors contributed equally. All authors contributed to the article and approved the submitted version.

## Conflict of Interest

VP and FM were employed by the company Refuge Biotechnologies. AC was employed by ESSA Pharmaceuticals.

The remaining author declares that the research was conducted in the absence of any commercial or financial relationships that could be construed as a potential conflict of interest.
